# Evaluation of the Fractured Surface of Five Endodontic Rotary Instruments: A Metallurgical Study

**DOI:** 10.22037/iej.2016.6

**Published:** 2016

**Authors:** Mohsen Aminsobhani, Mohamad Saleh Khalatbari, Naghmeh Meraji, Abdollah Ghorbanzadeh, Ehsan Sadri

**Affiliations:** a *Department of Endodontics, Dental School, AJA University of Medical Sciences, Tehran, Iran; *; b * Department of Endodontics, Dental School, Tehran University of Medical Sciences, Tehran, Iran; *; c * Material Science and Engineering Department, Sharif University of Technology, Tehran, Iran; *; d * Department of Physics, Science School, Central Tehran branch of Azad University, Tehran, Iran*

**Keywords:** Differential Scanning Calorimetry, Instrumentation, Nickel-Titanium, Root Canal Preparation, Scanning Electron Microscopy, Transition Temperatures, X-ray Diffraction

## Abstract

**Introduction:**

The aim of this study was to compare several metallurgic properties of Neoniti instrument with four other commonly used endodontic rotary files.

**Methods and Materials:**

Neoniti A1 (25/0.08), RaCe (25/0.06), Mtwo (25/0.06), Twisted file (25/0.06) and ProTaper Next X2 (25/0.06) were examined by differential scanning calorimetry (DSC) before and after heat treatment at 500^°^C. X-ray diffraction (XRD) was also performed on the specimens. Furthermore, scanning electron microscopy (SEM) and x-ray energy-dispersive spectrometric (EDS) analyses were carried out on randomly selected fractured files.

**Results:**

In SEM tests, dimpled ruptures, characteristic of ductile fracture, were seen in all evaluated cross sections of all files. The SEM results of all evaluated files were alike. EDS results revealed higher proportions of Nickel (Ni) rich intermetallic compounds in Neoniti; whereas, in all the other files the proportion of Titanium (Ti) rich precipitates was higher. DSC results indicated that the temperature present in the oral environment, the austenite phase existed in all files. Mtwo and RaCe files did not show austenite transformation in the temperature range evaluated in this study. Only Neoniti revealed rhombohedal phase (R-phase) transformation. After heat treatment. No significant difference was seen in the transformation temperatures of all evaluated files. XRD evaluations revealed that Neoniti contained both Ni-rich and Ti-rich precipitates. The amount of the martensite phase was higher in ProTaper Next.

**Conclusion:**

The metallurgic properties of Neoniti files were different from other evaluated rotary files. This file contained higher proportions of Ni-rich precipitates.

## Introduction

Manufactures have suggested various modifications in the NiTi file composition, geometry, heat treatment processes and so on*.* [1-5] as a solutions for their unexpected failures that occur happen without any visible signs of permanent plastic deformation [6]. Therefore, comparing different files with different modifications has always been a case of study [1, 7-12]. However, a metallurgical point of view regarding precipitates and intermetallic compounds which play an important role in their properties has been less noticed. Shen *et al.* [9] showed the presence of austenite phase in room temperature in NiTi files. X-ray energy-dispersive spectroscopic (EDS) results revealed the presence of titanium-rich inclusions with a relative composition of Ti_2_Ni [9]. Alapati *et al.* [13] concluded that the presence of some oxide particles, produced during manufacturing processes, are responsible for fracture of the evaluated rotary files and are the main locations of crack propagation.

**Figure 1:**
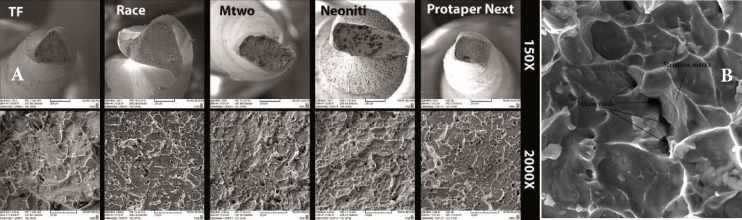
*A*) SEM images of fractured surfaces of evaluated files with 150× and 2000× magnification, *B*) SEM image of an Mtwo specimen revealing striation marks and micro-voids

Neoniti (Neolix, Châtres-la-Forêt, France) is a newly introduced NiTi rotary system manufactured with wire cut electrical discharge machining (EDM) process. High degree of accuracy, low residual stresses and better surface finishing are some advantages of this technique [14]. It has been shown that many characteristics of alloys are influenced by undergoing EDM process such as fatigue resistance [15] and surface hardness [16]. Moreover, the manufacturer claims that these files undergo appropriate heat treatment that leads to high flexibility. Its higher resistance to cyclic fatigue has been confirmed in one study [17].

M-Wire alloys are also produced by applying a series of heat treatments to NiTi wire blanks. These alloys have higher resistance to cyclic fatigue [5, 18-20]. ProTaper Next (X1; 17/0.04, and X2; 25/0.06, X3; 30/0.07, X4; 40/0.06 and X5; 50/0.06) (Dentsply, Maillefer, Ballaigues, Switzerland) is a newly marketed rotary file system made of M-Wire NiTi with a rectangular cross-sectional design. It has a progressive and regressive percentage taper on a single file. 

Twisted file (25/0.04, 25/0.06, 25/0.08, 25/0.10, 25/0.12, 30/0.06, 35/0.06, 40/0.04 and 50/0.04) (SybronEndo, Orange, CA, USA) is manufactured by twisting a NiTi wire with rhombohedal phase (R-phase) crystalline structures created by heating and cooling. Afterwards, it is heated and cooled again to convert back into the austenite crystalline structure while maintaining its new shape [21]. Studies have shown that this file has higher resistance to failure [17, 22, 23]. 

Since the microstructure and phase transformation behavior determine the mechanical properties of NiTi alloys, this study evaluates several metallurgic properties of five endodontic rotary files [Neoniti A1 (25/0.08), RaCe (25/0.06), Mtwo (25/0.06), Twisted file (25/0.06) and ProTaper Next X2 (25/0.06)] to investigate the reasons of file fracture and probable methods for prevention.

## Materials and Methods

Five types of rotary files with a similar tip size (*n*=60) were evaluated in this study: Neoniti A1 (25/0.08, Neolix Sas, Châtres-La-Forêt, France), RaCe (25/0.06, FKG Dentaire, La-Chaux-de Fonds, Switzerland), Mtwo (25/0.06, VDW, Munich, Germany), Twisted file (25/0.06, SybronEndo, Orange, CA, USA) and ProTaper Next X2 (25/0.06, Dentsply Maillefer, Ballaigues, Switzerland).

***SEM and EDS evaluations***

Fifteen fractured fragments obtained from our previous study [17] were randomly selected from files that had undergone cyclic fatigue testing in three types of trajectories each measuring 1.5 mm in width, 20 mm in length and 2.5 in depth into chrome plating polished 316 L stainless steel blocks in our previous study. The trajectory designs were as follows. Group A: a straight cervical segment measuring 5.29 mm with an arc length of 9.42 mm, a curvature radius of 6 mm, a straight apical segment measuring 5.29 mm and the arc in the middle portion of the canal; group B: a straight segment measuring 7.44 mm with an arc length of 12.56 mm, a radius of 6 mm, and the arc in the apical portion of the canal; group C: a straight cervical segment measuring 10.58 mm with an arc length of 9.42 mm, a curvature radius of 6 mm, and the arc located in the apical portion of the canal.

The fractured fragments were then examined under a scanning electron microscope (SEM) (Vega, Tescan-Lmu, Usa) to determine the characteristics of fracture. Afterwards, qualitative EDS was used to determine the average amounts of the nickel (Ni), titanium (Ti) and other elements in the rotary instruments. Additionally, if a spot with different contrast was identified during SEM evaluations it would also be analyzed by EDS.

**Figure 2:**
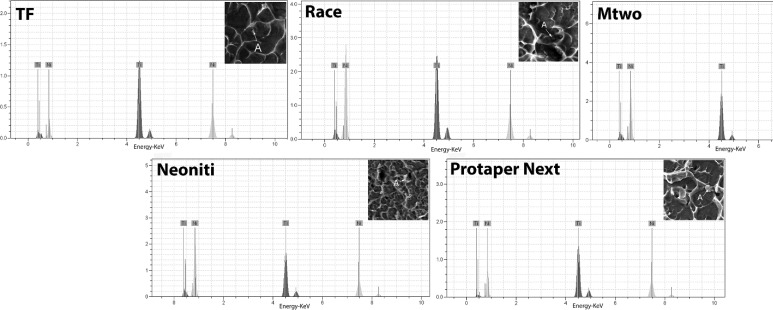
X-ray energy-dispersive spectrometric (EDS) results of evaluated files

***Differential scanning calorimetry (DSC)***

One randomly selected unused file from each type was analyzed by DSC (DSC Q 100-USA). DSC analysis was conducted in temperatures ranging from -50^°^C to +200^°^C by the use of a liquid nitrogen cooling accessory to achieve sub ambient temperatures. The linear heating/cooling rate was a standard 10^°^C/min. The martensitic transformation-starting and transformation-finishing points (M_s_, M_f_) and reverse transformation-starting and transformation-finishing points (A_s_, A_f_) were determined. 

Subsequently, the specimens were heat treated at 500°C for 1 h in argon atmosphere and once cooled, were analyzed again. 

***X-ray diffraction***

X-ray diffraction (XRD) (X’Pert Pro MPD; PANalytical BV, Almelo, The Netherlands) was performed to identify phases in unused files and complete the DSC results. Eight specimens from the shaft of each file type were adhered together by acrylic resin and were then ground by using silicon carbide sandpaper with decreasing particle sizes of 400, 500, 800, 1000, 1200, 1500 and 2000 grit, respectively. Subsequently the surface of the specimens was polished, washed with ethyl alcohol and analyzed by XRD.

## Results

***SEM***

Figure 1A shows the SEM results for all files. The cross sections of all evaluated files were alike. Dimpled ruptures, characteristic of ductile fracture, were seen in all evaluated cross-sections. Furthermore, numerous micro-voids were seen in the ductile fracture cross sections. 

Only in one of the evaluated Mtwo specimens, striation marks in conjunction with micro-voids were seen (Figure 1B) which was indicative of the occurrence of brittle fracture in that specimen [7]. No notable difference was seen in the SEM results of each file type when used in different canal types; therefore, indicating no difference in the fracture mechanism of each file when used in three different curvatures. Only the stress concentration site differed in different curvatures. 

***EDS***

EDS evaluation of all files confirmed their chemical composition to consist of nickel and titanium (Figure 2). In RaCe, Mtwo and Twisted file specimens the titanium-rich precipitate content was higher whereas, for Neoniti files, the proportion of nickel-rich precipitates was higher. 

***DSC***

**Figure 3:**
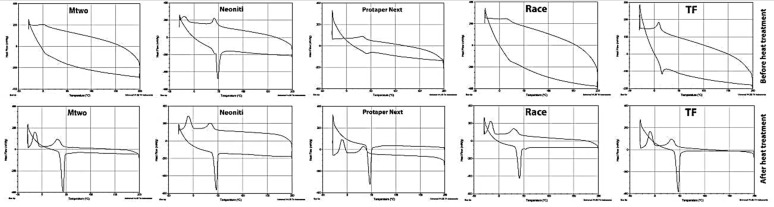
Differential scanning calorimetric (DSC) plots before and after heat treatment. Heating (lower) and cooling (upper) curves are shown

Figure 3 represents DSC plots for both the heating and cooling cycles of different types of instruments before and after heat treatment. In these curves, endothermic peaks represent the austenite transformation and exothermic peaks represent the martensitic transformation. After heat treatment the transformation temperatures of all evaluated files were similar and no significant difference was seen. The A_s_ increased in all files. 

The A_s_ temperatures for all evaluated rotary instruments were in a manner that in the mouth temperature the austenite phase existed. Mtwo and RaCe files did not show austenite transformation in the temperature range evaluated in this study (Tables 1 and 2). Only Neoniti revealed R-phase transformation.

***X-Ray Diffraction***

**Figure 4:**
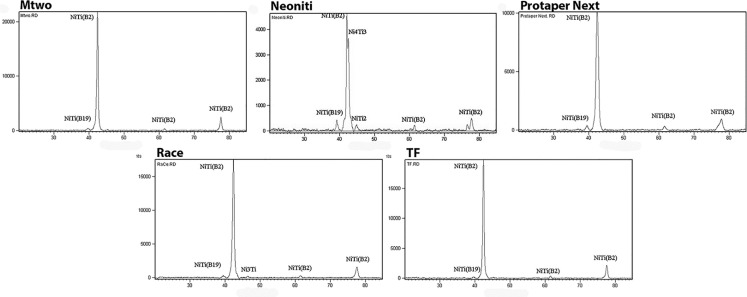
X-ray diffraction (XRD) patterns of the evaluated files

Figure 4 shows the XRD patterns of the evaluated files. The predominant peaks seen in the XRD evaluation of all files were the B2 (austenite) and B19ʹ (martensite) phases. The intensity of these peaks differed between Mtwo, RaCe, ProTaper Next and Twisted file. The amount of the martensite phase was higher in ProTaper Next compared to the other three files. In Mtwo, ProTaper Next and TF precipitates such as NiTi_2_, Ni_4_Ti_3_ and Ni_3_Ti were not seen whereas peaks representing Ni_3_Ti were seen in RaCe. 

Neoniti showed different results compared to the other four evaluated files. Both Ni-rich and Ti-rich precipitates were seen in this file. 

## Discussion

In this study the metallurgic properties of five endodontic rotary files were evaluated.

In the SEM evaluation dimple ruptures and micro-voids were seen in all fractured segments of all evaluated files. In ductile fractures, micro-voids are produced within the metal and the process of nucleation, growth and micro-void coalescence ultimately weakens the metal and results in fracture. Plastic deformation due to slipping also contributes to ductile fracture. The fracture surface resulting from these two processes is generally characterized by a dull dimpled surface [7]. The shape and slope of the dimples may indicate the type of load applied as well as the origin of the fracture [24].

Elongated dimples in the file cross sections indicate that tearing and shearing forces are responsible for failure [24]. These dimples may be produced either in the course of the manufacturing process or during deformation [13]. Elongated dimples were seen in all specimens.

Furthermore, striation marks were not seen in the cross section of any of the evaluated files except for one of the Mtwo specimens. The presence of striation marks is characteristic of fatigue failure [7]. It has been shown that fatigue failure in NiTi rotary files leads to ductile fracture [25]. Nevertheless, absence of these marks in the majority of the evaluated files shows that either ductile fatigue fracture [25] or fracture occurred due to overloading in one rotation cycle [26].

SEM evaluations showed that the cross section of files used in different canal types did not differ. This indicates that the fracture mechanism of each file when used in different canal types does not differ and the only difference is the stress concentration site which is the initiation point of failure.

EDS evaluations revealed that only in Neoniti the percentage of nickel-rich precipitates is higher; whereas, in the other evaluated files the percentage of titanium-rich precipitates is more prominent. 

As shown in Figure 3, no austenite transformation was seen in Mtwo and RaCe files. The amount of work hardening may be responsible for this phenomenon. The presence of impurities and work hardening are the main reasons for the absence of transformation [27]. In fact impurities and work hardening cause excessive pile up of dislocations and other crystal imperfections. These imperfections act as a barrier against the occurrence of the transformation [27]. 

**Table 1:** Transition temperatures for the files before heat treatment

**Transition Temperature**	**A**_s_	**A**_f_	**M**_s_	**M**_f_	**R**_s_	**R**_f_
**Mtwo**	32.45	52.5	-8.3	-32	47	29.16
**Neoniti**	37.5	52.5	2.5	-20.83	47.5	19.95
**ProTaper Next**	37.8	54.16	2	-20.8	45.87	16.6
**Race**	31.25	49	5.9	-29.16	41.6	15
**TF**	37.9	58.37	2.5	-27.5	44.75	20.83

**Table 2.** Transition temperatures for the files after heat treatment

**Transition Temperature**	**A**_s_	**A**_f_	**M**_s_	**M**_f_	**R**_s_	**R**_f_
**Mtwo**	-	-	10	-8.3	-	-
**Neoniti**	37.5	52.5	2.5	-20.83	47.5	19.95
**ProTaper Next**	37.8	54.16	2	-20.8	45.87	16.6
**Race**	-	-	28	8.3	-	-
**TF**	12.5	23.6	22.5	-8.3	-	-

R transformation is another difference in these curves which was only seen in Neoniti. R transformation has also been reported in similar rotary files such as Hyflex EDM [28], which is also manufactured by EDM, ProTaper Gold files [29] and Vortex Blue files [30] which have no rebound effect after unloading as seen in Neoniti files [29]. In Ni-rich NiTi alloys an intermediate R-phase may exist between the austenite to martensitic transformation [27, 31]. Some of the properties of the R-phase are: superior fatigue resistance, narrow hysteresis and allowing stress relaxation [32]. In general, anything that delays the martensitic transformation leads to R transformation. Some of these situation are created by disordered dislocations in the alloy texture caused by cold working and post annealing in temperatures between 400˚C and 500˚C, solid solution and post aging in temperatures between 400˚C and 500˚C in Ni-rich NiTi alloys and adding a third element in NiTi alloy system (such as Al or Fe) [33-35]. Schryvers *et al.* [36] suggested that coherent Ni_4_Ti_3_ precipitates in Ni-rich NiTi alloys result in Ni depletion in the matrix and generation of strain fields around the precipitates. These strain fields lead to the formation of the R-phase.

The A_f_ temperature of Neoniti and ProTaper Next was higher than their working temperature, indicating the presence of stable martensite phase as seen in ProTaper Gold [29]. High A_f_ temperature has also been reported in Hyflex EDM [28] and ProTaper Gold files [29].

Figure 3 shows DSC curves after the heat treatment. Heat treatment was done in 500˚C for 1 h in argon atmosphere on all specimens. After heat treatment the transformation temperatures of all evaluated files were similar and no significant difference was seen. It can be suggested that the initial composition of all the rotary files were relatively identical and the noted differences are due to mechanical working and heat treatment cycles during the manufacturing process. 

As shown in Table 2 after heat treatment the A_s_ temperatures increased in all files which indicates the elimination of defects caused by work hardening and redistribution of precipitates after heat treatment. 

Alapati *et al.* [2] evaluated the influence of heat treatment on transformation temperatures. They suggested that after heat treatment in 500˚C, two peaks corresponding to martensite-to-R-phase transformation at lower temperature and R-phase-to-austenite transformation at higher temperature can be observed. But even after heat treatment the cooling curves still contain a single peak. By increasing the heat treatment temperature process to 600˚C, two stage transformations can be seen in both cooling and heating curves. They also suggested the absence of R transformation in specimens without heat treatment is due to cold working during the manufacturing process [2].

Figure 4 shows XRD patterns of evaluated files. All these patterns show the main peaks of NiTi alloy. The most important peaks were the ones related to austenite (B2) and martensite (B19ʹ) phases. The results of Neoniti were different from that of the others. The only difference between the other files was the intensity of the martensite peaks. The intensity of the martensite peaks in ProTaper Next files was more than that of the other files (Figure 4). Thus ProTaper Next files contain more martensite phase. No intermetallic phases such as NiTi_2_, Ni_3_Ti and Ni_4_Ti_3_ was seen except for the case of RaCe files which demonstrated a Ni_3_Ti peak. The pattern of Neoniti was completely different and both Ni-rich and Ti-rich compounds were seen.

The structure of Ti-rich secondary phases in NiTi is not complicated. As it can be understood from the NiTi phase diagram, the amount of excessive Ti in the texture in maximum temperature is less than 5 atm%. Formation of precipitates such as NiTi_2_ in Ti-rich alloys is inevitable. This kind of precipitate can usually be seen in grain boundaries. Transition temperatures in Ti-rich NiTi are not dependent on these precipitates [37].

Formation of secondary phases in Ni-rich NiTi is more complicated. According to the NiTi phase diagram, Ni solubility in NiTi is strongly dependent on the annealing temperature. The solubility of Ni decreases from 7 atm% in 1118˚C to 0 atm% in 630˚C. So Ni saturated texture is expected in high temperatures. Transition temperatures, mechanical properties and shape memory properties are influenced by secondary phases in Ni-rich NiTi alloys. Texture phase and stress fields around precipitates are dependent on the annealing temperature [38].

Ni_3_Ti_2_ and Ni_3_Ti precipitates are usually homogeneous in the texture phase. Therefore, the presence of these precipitates usually cannot influence the shape memory effect in Ni-rich NiTi alloys. Contrarily, Ni_4_Ti_3_ precipitates can deposit in the texture B2 phase homogeneously, semi-homogeneously or heterogeneously and thus can affect shape memory properties [38, 39]. Another result of the presence of Ni_4_Ti_3_ precipitates is formation of the R phase [36].

It can be concluded that higher transition temperatures in Neoniti files may be caused by intermetallic phases. But to investigate the exact influence of these precipitates on mechanical properties, micro-structure and distribution of the phases should be studied. Furthermore, similar evaluation of files undergoing torsional failure is also suggested.

## Conclusion

The metallurgic properties of Neoniti files were different from other evaluated files. This file contains higher proportions of Ni-rich precipitates. Differences seen in the mechanical properties of this file may be due to these differences. It should be noted that the initial composition of all the rotary files were relatively identical and the differences are caused by mechanical working and heat treatment cycles during the manufacturing process.
